# Cardio-Hypothalamic-Pituitary Coupling during Rest and in Response to Exercise

**DOI:** 10.3390/e24081045

**Published:** 2022-07-29

**Authors:** Nathaniel T. Berry, Christopher K. Rhea, Laurie Wideman

**Affiliations:** Department of Kinesiology, University of North Carolina at Greensboro, Greensboro, NC 27412, USA; ckrhea@uncg.edu (C.K.R.); l_widema@uncg.edu (L.W.)

**Keywords:** growth hormone, heart rate variability, nonlinear dynamics, physiologic coupling

## Abstract

The objective of this study was to examine cardio hypothalamic-pituitary coupling and to better understand how the temporal relations between these systems are altered during rest and exercise conditions. An intensive within subjects study design was used. Seven adult males completed two visits, each consisting of either a 24 h period of complete rest or a 24 h period containing a high-intensity exercise bout. An intravenous catheter was used to collect serum samples every 10 min throughout the 24 h period (i.e., 145 samples/person/condition) to assess growth hormone (GH) dynamics throughout the 24 h period. Cardiac dynamics were also collected throughout the 24 h period and epoched into 3 min windows every 10 min, providing serial short-time measurements of heart rate variability (HRV) concurrent to the GH sampling. The standard deviation of the normal RR interval (SDNN), the root mean square of successive differences (rMSSD), and sample entropy (SampEn) was calculated for each epoch and used to create new profiles. The dynamics of these profiles were individually quantified using SampEn and recurrence quantification analysis (RQA). To address our central question, the coupling between these profiles with GH was assessed using cross-SampEn and cross-RQA (cRQA). A comparison between the epoched HRV profiles indicated a main effect between profiles for sample entropy (*p* < 0.001) and several measures from RQA. An interaction between profile and condition was observed for cross-SampEn (*p* = 0.04) and several measures from cRQA. These findings highlight the potential application of epoched HRV to assess changes in cardiac dynamics, with specific applications to assessing cardio hypothalamic-pituitary coupling.

## 1. Introduction

The inherently complex and intricately connected regulatory mechanisms associated with physiologic control span biologic subsystems and often display fractal and multifractal characteristics in structure and time. The fractal and multifractal time series from many of these systems have been a focal point of research for several decades and include cardiac dynamics [1,2,3,4,5,6], human locomotion [7,8,9,10,11,12,13], and postural control [14,15,16,17,18]. These efforts are based on the seminal findings in this area that demonstrate variability and complexity of physiologic phenomena representing the adaptability of the underlying systems [19,20,21,22] and that traditional statistics, such as averages, may not accurately represent the continuum from health to disease [19,23].

The normal heartbeat was one of the first physiologic phenomena to be described as a fractal process [19,23] and since then, heart rate variability (HRV) has been a popular, and informative, noninvasive tool to assess and quantify cardiac autonomic regulation. Heart rate variability is defined as the time between consecutive RR intervals and can be analyzed through a variety of statistics, providing insight into the physiological interplay between the parasympathetic and sympathetic nervous systems [24,25]. Most commonly, HRV is assessed through either 24 h or short-time (~3–5 min) measurements that are subsequently used to compare groups and/or in response to some perturbation. Throughout the 24 h period, both parasympathetic and sympathetic inputs contribute to changes in the standard deviation of the normal RR interval (SDNN), while the root mean square of successive differences (rMSSD) primarily represents regulation from the parasympathetic nervous system [25]. Short-time HRV measurements provide an assessment of cardiac autonomic control within a specific window and provide different information about the overall status of the system compared to a 24 h measurement. Within these short-time recordings, SDNN is heavily influenced by the sympathetic nervous system and respiratory sinus arrhythmia while rMSSD is predominantly representative of vagally mediated inputs [24,25].

During rest, depressed values of HRV are associated with an increase in sympathetic activity whereas higher values of HRV (greater variability) typically represent an increase in parasympathetic activity [24,25]. The addition of nonlinear dynamics in HRV research has provided information about cardiac regulatory behavior that is both different and unobtainable from traditional measures of variability (e.g., standard deviation) [19,23]. Importantly, most evidence suggests that too little or too much complexity within these systems represents maladaption and reduced adaptability [19,20,21,22]. For instance, chronic disease has been shown to reduce measures of nonlinear HRV while increased nonlinear HRV following myocardial infarction is associated with increased mortality [26]. While changes in the complexity within systems have been shown to represent the manifestation and presence of disease [19,23], disease also alters the coupling between biological subsystems [27,28,29].

In addition to the heart, the autonomic nervous system innervates the hypothalamus at the paraventricular nucleus. The hypothalamus is one of the key regulators of physiologic control and the paraventricular nucleus is essential to this regulation [30]. Growth hormone-releasing hormone (GHRH) and somatostatin are released from the hypothalamus and, in combination with other peripheral feedback signals, these hormones regulate growth hormone (GH) output from the anterior pituitary [31]. With a half-life of ~16–19 min [31,32,33] and pulsatile secretory dynamics, changes in GH concentration occur rapidly. These rapid changes in GH concentration provide information about the overall status of the system, with differentiable effects of gender and age, as well as nutrition, sleep, body composition, health, and fitness [31,32].

Changes in GH output and secretory dynamics [31] often mirror changes observed in HRV indices [24,25,34] across the lifespan and with the progression of disease. Similarly, both GH and heart rate react very specifically to various perturbations, including exercise. Investigation into the autonomic nervous system and hypothalamic-pituitary axis, independent of the other, are plentiful, but a direct exploration of how these two systems interact is less common [35,36,37,38].

An obvious question is whether these observations are happenstance or if these systems couple in more acute and immediate measures of time. A better understanding of the relations between biologic subsystems, particularly the relations between markers requiring noninvasive versus invasive measurement, have clinical and practical relevance in exercise, sport, military operations, and clinical settings. For instance, understanding how these systems couple during rest and in response to an acute perturbation may provide a method of better-characterizing disease progression, quantifying risk, or the potential to use the non-invasive measurements to estimate or predict underlying physiologic responses that would otherwise require invasive and time prohibitive testing. An immediate challenge is a discrepancy in the time scales of potential markers (e.g., GH and HRV).

The overall objective of this study is to examine cardio hypothalamic-pituitary coupling and to better understand how the temporal relations between these systems are altered during rest and exercise conditions. We first examine methods of processing a continuous RR-recording into discrete short-time assessments of HRV (epoched HRV) to address issues of timescale invariance and explore the physiological meaning of these time series. We subsequently use these data to examine the time-dependent and temporal relations between these measures of cardiac dynamics with growth hormone. We hypothesized that each of the epoched HRV profiles would couple with GH output during rest and exercise, but that exercise would increase this coupling. Further, we considered that there may be a difference in the degree of coupling between each of the epoched HRV profiles and GH output, providing different information about the regulation of these systems with implications for practical applications and future directions.

## 2. Materials and Methods

**Figure 1:**
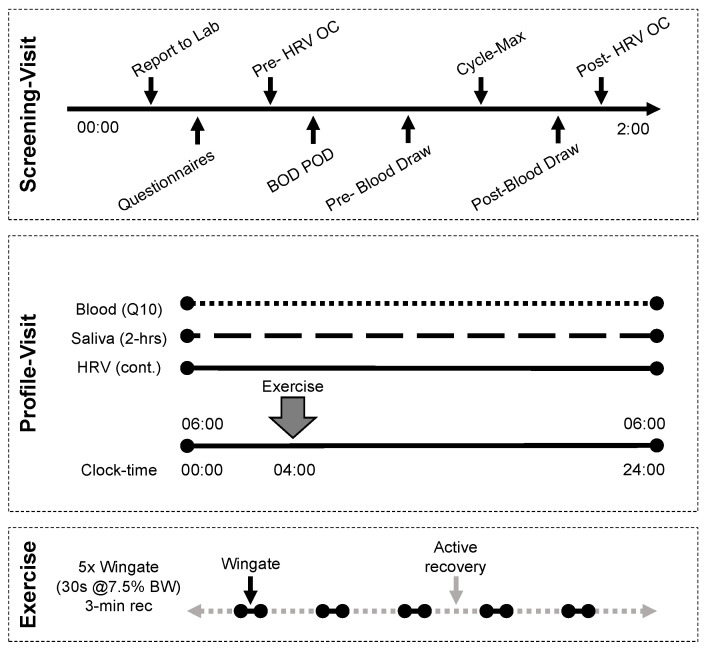
Study design. Each participant completed two profile visits, separated by a minimum of 8 weeks. Screening visits were performed no less than 48 h prior and no more than 14 days from the profile visit.

An intensive within subjects study design was used. The study design is provided in Figure 1 and additional detail on the methods is provided in the respective sub sections. Healthy adult males (N = 7) were included in this study. Each participant completed a rest and exercise condition separated by a minimum of 8 weeks. A screening visit was conducted prior to each of the rest and exercise conditions (profile visits). Demographic information was collected during each screening visit and each profile visit consisted of a 24 h admission with serum collected every 10 min (Q10) and RR-intervals collected continuously. The exercise condition consisted of a high-intensity interval workout completed on the cycle ergometer.

### 2.1. Sample

All individuals were healthy adult males who regularly participated in moderate-vigorous exercise (≥4 days/week). All subjects were free of any known metabolic, cardiovascular, or pulmonary disease and had a body composition <18% fat. Exclusion criteria included acute or chronic health conditions, medications for cardiovascular or metabolic disease, mental health, endocrine, infectious conditions, a history of cancer, or additional conditions that would have jeopardized participant safety were excluded from this study.

### 2.2. Screening Visit

Body composition was assessed with COSMED’s BOD POD (body fat, BF; fat-free mass, FFM). Training history was assessed via questionnaire and used to ensure that activity levels had not changed drastically between the two admissions. To conclude the screening visit, participants completed a ramp test (100 W + 25 W/min) on a cycle ergometer to volitional fatigue (Lode Excalibur Sport). Breath-by-breath oxygen uptake was collected (Parvo Medics TrueOne 2400) to assess maximal oxygen uptake (VO_2max_).

### 2.3. Profile Visit

The profile visits were completed no less than 48 h and no more than 14 days following each of the screening visits. Each profile visit consisted of either a 24 h period of complete rest or a 24 h period containing a high-intensity exercise bout. The order of the rest and exercise conditions was randomly assigned and separated by a minimum of 8 weeks due to limits on blood volume collection. Participants reported to the laboratory at ~05:30 to begin the 24 h admission at 06:00. An intravenous catheter was placed in either the radial or antecubital vein and [3 mL] serum samples were collected every 10 min (Q10) throughout the entire 24 h period (i.e., 145 samples/person/condition). A Polar HR monitor (V800) was used to collect RR intervals continuously. Subjects were permitted to ambulate in the laboratory but were not permitted to exercise except during the prescribed high-intensity interval session. To standardize macronutrient intake prior to and immediately following the exercise bout (at 10:00), subjects ate breakfast ~07:30 and were restricted to water between 08:00–10:30. All subjects ate lunch ~13:00, and dinner ~20:00. All food and beverages consumed by the participants were detailed in a dietary log and participants were asked to consume foods of similar macronutrient composition during the second profile visit. Participants were permitted to go to bed at their discretion, with mandatory lights-out at 23:00.

### 2.4. Exercise Protocol

Following a 5 min warmup (≤50 watts), participants completed a high-intensity exercise workout consisting of five 30 s maximal efforts on the cycle ergometer. Each effort was separated by a 3 min active recovery period. Force applied to the flywheel was standardized at 7.5% of body mass (kg) for each subject.

### 2.5. Biological Sample Collection and Analysis

**Figure 2:**
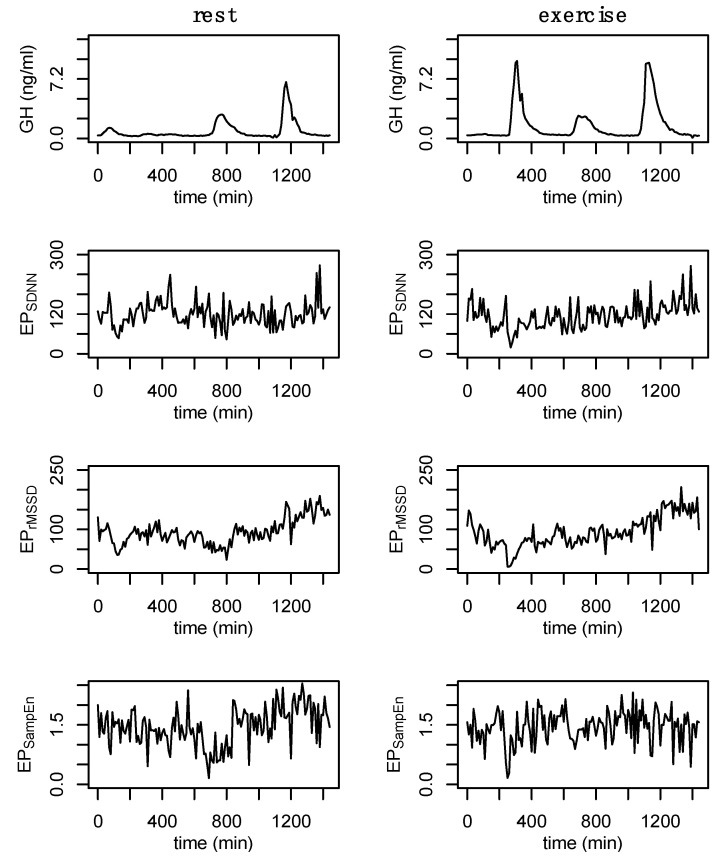
Example data from a single participant. Growth hormone (GH) during rest and exercise followed by the epoched HRV profiles (EP_SDNN_, EP_rMSSD_, and EP_SampEn_) from the after-3 (a3) sampling method. These new data were generated by analyzing 3 min windows from the 24 h RR recording every 10 min. Data are from subject 3.

The intravenous catheter was connected to a normal saline drip with a keep-vein-open protocol to maintain line patency (20–30 mL/h). Blood was collected in a serum separator tube through a closed system, and participants were volume-repleted with the waste and a ~3 mL bolus of normal saline. Samples were allowed to clot for 20–40 min and were then centrifuged for 12 min at 3000 rpm Serum was aliquoted into 1.5 mL storage tubes and frozen at −80 °C until assayed. Growth hormone (GH) was assayed using commercially available enzyme-linked immunosorbent assays (RayBiotech, Peachtree Corners, GA, USA). Growth hormone output across the entire 24 h period, daytime hours (06:00–06:00), nighttime hours (10:00–6:00), and exercise hours (10:00–12:00) were calculated. Similarly, the peak exercise (10:00–12:00) and nighttime (22:00–06:00) GH concentrations were measured. Nadir GH concentration across the entire 24 h was also assessed. An example of the 24 h GH profile during rest and exercise conditions is provided in Figure 2.

### 2.6. RR-Recordings

The 24 h recordings were pre-processed following guidelines set forth by the 1996 Task Force for HRV [24] using RHRV [39]. Measures of 24 h HRV included the standard deviation of the normal RR-intervals (SDNN), the root mean square of successive differences of the normal RR-interval (rMSSD), and sample entropy (SampEn). Sample entropy of the 24 h recording was calculated using an embedding dimension, *m*, of *m* = 2 and a tolerance/radius, *r*, of *r* = 0.2σ [denoting a percentage of the standard deviation of the entire time series]. These parameters are commonly used for HRV analyses [40,41,42,43,44] and were chosen for consistency to existing literature.

### 2.7. Epoched RR-Recordings

**Figure A1:**
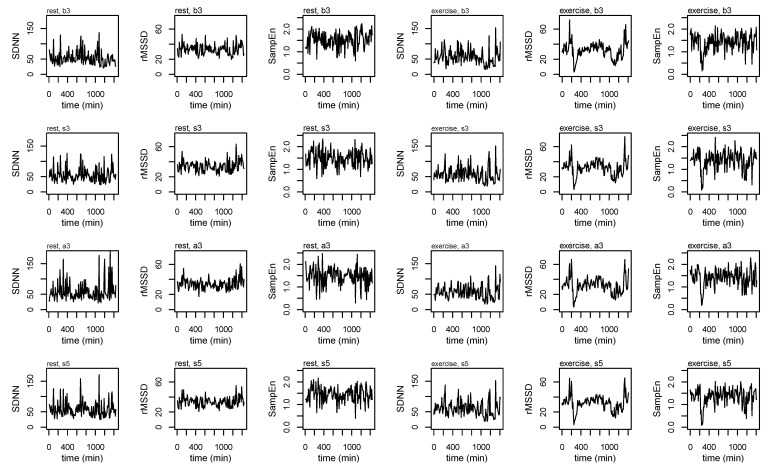
Heart rate variability (HRV) profiles for a single subject across each of the epoching methods and during rest and exercise conditions.

A custom algorithm was used to process each of the cleaned 24 h RR recordings and analyze various short-time epochs taken serially throughout the 24 h period. Based on other assessments embedded into this protocol, all epochs reference a 10 min interval. For example: before-3 (b3) refers to minutes 00:7:00–00:10:00, 00:17:00–00:20:00, etc.; split-3 (s3) refers to minutes 00:08:30–00:11:30, 00:18:30–00:21:30, etc.; after-3 (a3) refers to minutes 00:10:00–00:13:00, 00:20:00–00:23:00, etc.; and split-5 (s5) refers to minutes 00:07:30–00:12:30, 00:17:30–00:22:30, etc. (in the format HH:MM:SS). The SDNN, rMSSD, and sample entropy of each of these epochs were calculated and these values were used to create separate time series (EP_SDNN_, EP_rMSSD_, and EP_SampEn_, respectively) that were used to assess changes in short-time measures of HRV throughout the 24 h period. An example of each of these time series, for a single individual, at rest and exercise is provided in Appendix A (Figure A1). An example showing a comparison of the epoched HRV profiles from the a3 processing method at rest and exercise is provided in Figure 2.

### 2.8. State-Space Reconstruction

**Figure 3:**
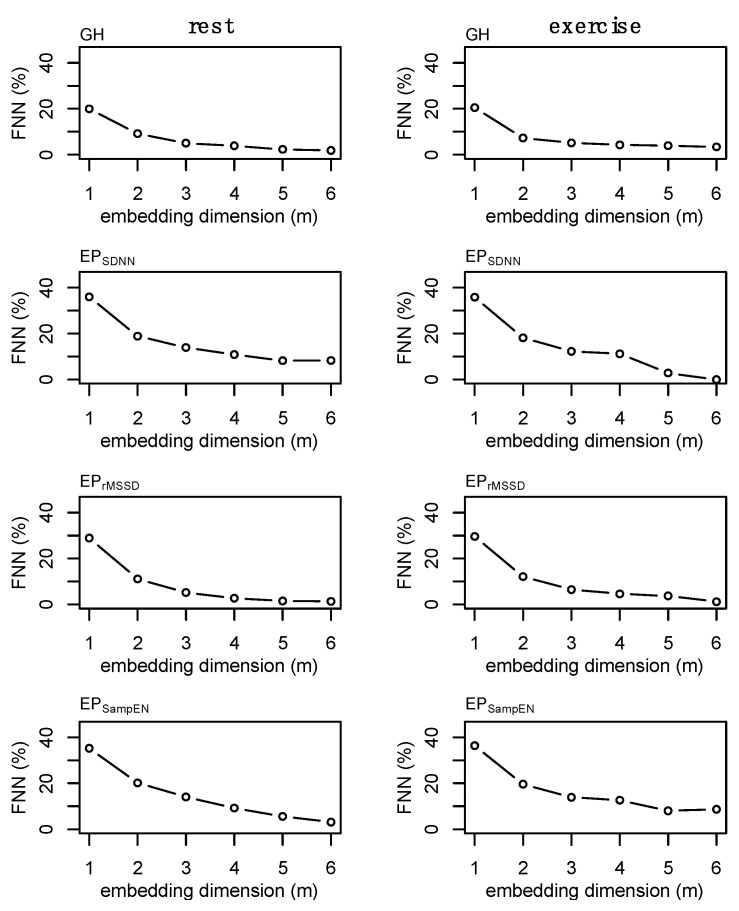
Example of the false nearest neighbors (FNN) calculations for the epoched HRV profiles (i.e., EP_SDNN_, EP_rMSSD_, and EP_SampEn_) and growth hormone (GH) profiles and during fest and exercise. These plots were generated for each subject and a single time-delay was chosen for each time series for subsequent analyses. Values for GH (*m* = 2), EP_SDNN_ (*m* = 2), EP_rMSSD_ (*m* = 2), and EP_SampEn_ (*m* = 2) were chosen. Note: final values were chosen based on consideration of all data, not solely from those presented above. Data are from subject 3.

**Figure A2:**
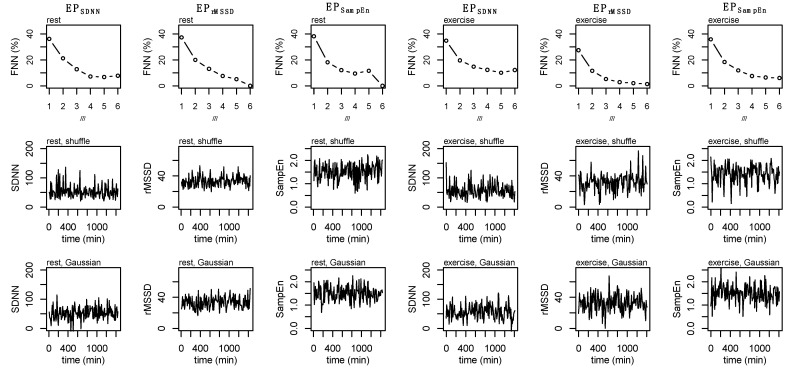
False Nearest Neighbors (FNN, **top row**), shuffle surrogate data (**middle row**), and Gaussian surrogate data (bottom row) for EP_SDNN_, EP_rMSSD_, and EP_SampEn_ during rest (**first three columns**) and exercise (**second three columns**).

State space reconstruction for GH and each of the epoched HRV profiles was performed based on considerations from data of all individuals. Time-delay, *L*, was determined through average mutual information and selection was based on the first value to decay below 1/e of the value at 0 lags. A time-delay of *L* = 2 was chosen for GH while *L* = 1 was selected for each of the epoched HRV profiles (i.e., EP_SDNN_, EP_rMSSD_, EP_SampEn_). The embedding dimension was determined through the use of false nearest neighbors [45]. The false nearest neighbors algorithm is impacted by the presence of noise [45,46], and data length [47]. In the presence of noise, the percent false nearest neighbors often does not reach ~0% as it does in other systems [45]. Because there is no defined threshold for false nearest neighbors to optimally define the embedding dimension in systems with noise, we chose the lowest embedding dimension (*m* = 2) that balances a reduction in the percentage of false nearest neighbors without overly estimating the dimension of the time series. Examples of the false nearest neighbors estimations for a single individual are provided in Figure 3. Growth hormone sampled Q10 for an entire 24 h period (n = 145) is a relatively short dataset within the nonlinear dynamics literature, however, this is considered a large amount of data within the endocrine literature. A final value of *m* = 2 was chosen for GH, EP_SDNN_, EP_rMSSD_, and EP_SampEn_. Data length and computational limitations around choosing higher embedding dimensions may be particularly relevant in the case of EP_SampEn_ where the percentage of nearest neighbors remained high at *m* = 2—although there was a significant reduction from *m* = 1 (Figure 3, bottom row). An example of the false nearest neighbors output from each of the epoched HRV processing methods is provided in Appendix A (Figure A2).

**Figure 4:**
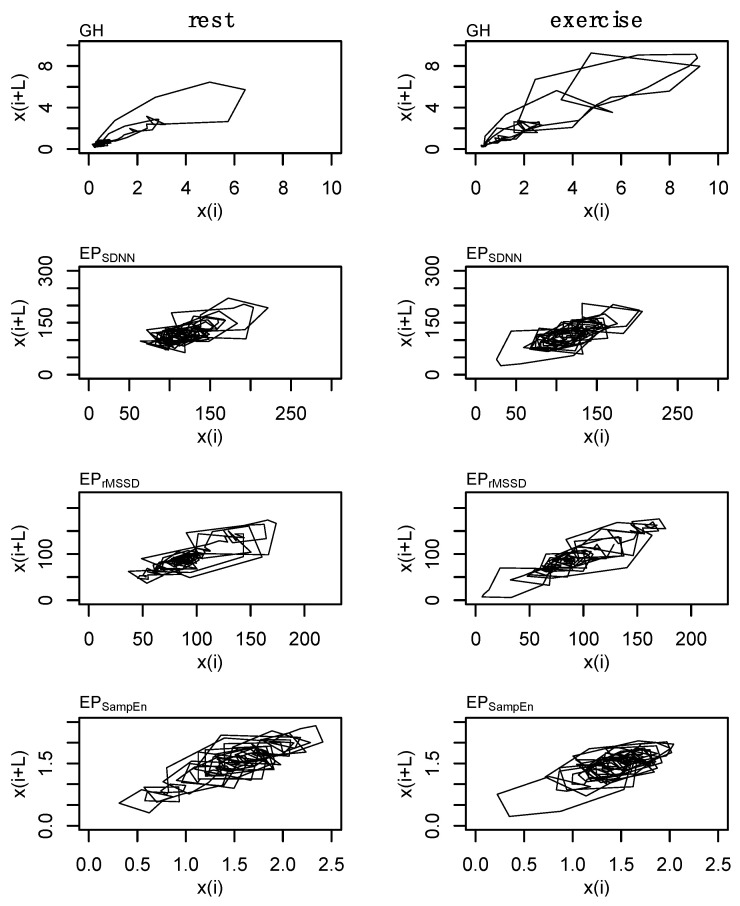
State space reconstruction for growth hormone (GH) and each of the epoched HRV profiles (i.e., EP_SDNN_, EP_rMSSD_, and EP_SampEn_). Data are from subject 3.

**Figure A3:**
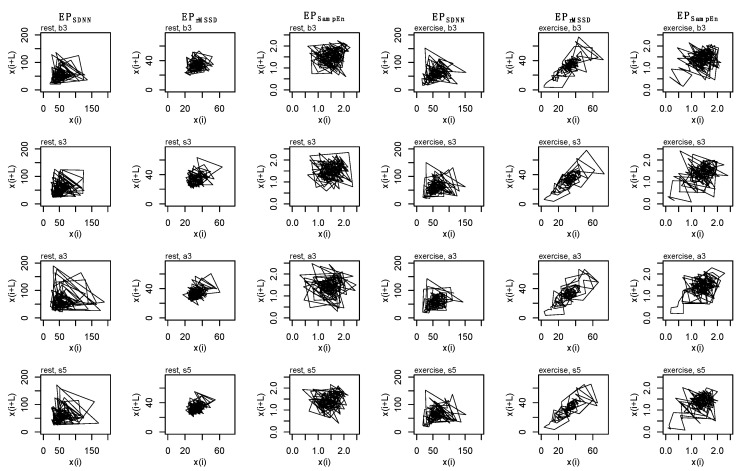
State space reconstruction for each epoched HRV profile (i.e., EP_SDNN_, EP_rMSSD_, EP_SampEn_), across each epoching method (i.e., before-3, b3; split-3, s3; after-3, a3; and split-5, s5) during rest (**first three columns**) and exercise (**second three columns**) conditions. Data are produced from a single subject.

These parameters were used in the state space reconstruction for each of these profiles (Figure 4). State space reconstruction was performed to provide a visual reference of the dynamics of GH and each of the epoched HRV profiles (i.e., EP_SDNN_, EP_rMSSD_, and EP_SampEn_) during rest and exercise conditions. An example of the state space reconstruction for each of the epoched HRV processing methods is provided in Appendix A (Figure A3).

### 2.9. Surrogate Data 

**Figure 5:**
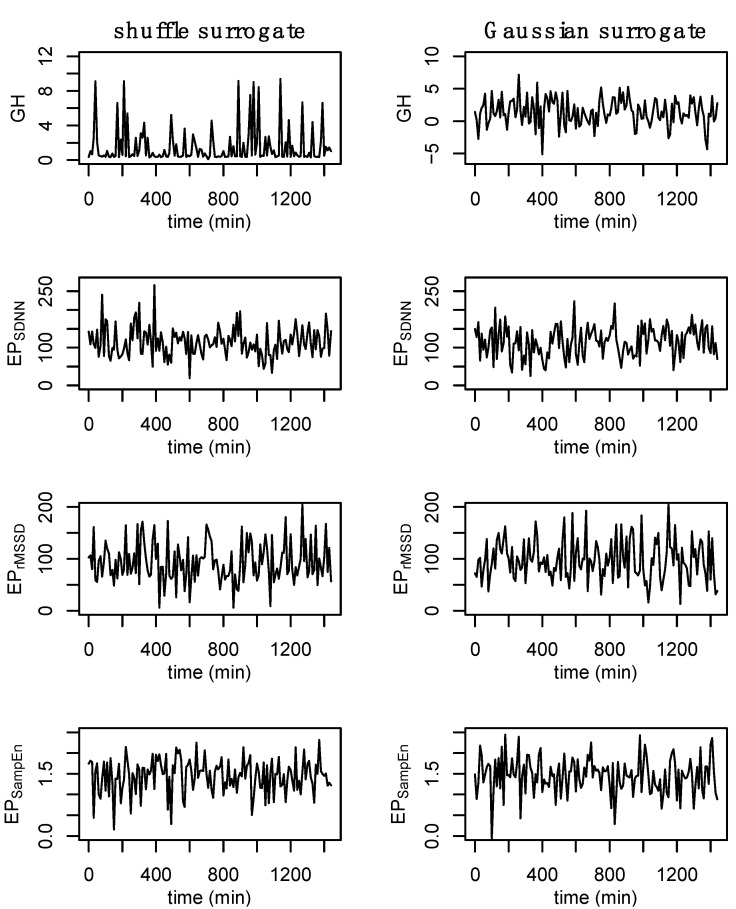
Example of surrogate data. Shuffle surrogate data were generated by randomly shuffling the data from each time series while Gaussian surrogate data were generated through the random sampling of values from a normal distribution with the mean and standard of the original time series. Consistent with previous figures, these data are from subject 3.

Surrogate data [48] were used to establish nonlinear and complex structure of the original time series. Shuffle and Gaussian surrogate data were generated for GH and each epoched HRV profile to examine whether these time series differ from random noise. Shuffle surrogate data were generated by randomly shuffling the data from each time series and Gaussian surrogate data were generated through random sampling of values from a normal distribution determined from the mean and standard of the original time series. The Hurst exponent [49,50], which provides a measure of long-term memory in a time series, was calculated for GH and each epoched HRV profile, and surrogate data, across all processing methods (i.e., b3, s3, a3, s5) to examine whether these data contain complex or nonlinear structure. An example of the surrogate data is provided in Figure 5.

### 2.10. Individual Dynamics

**Figure 6:**
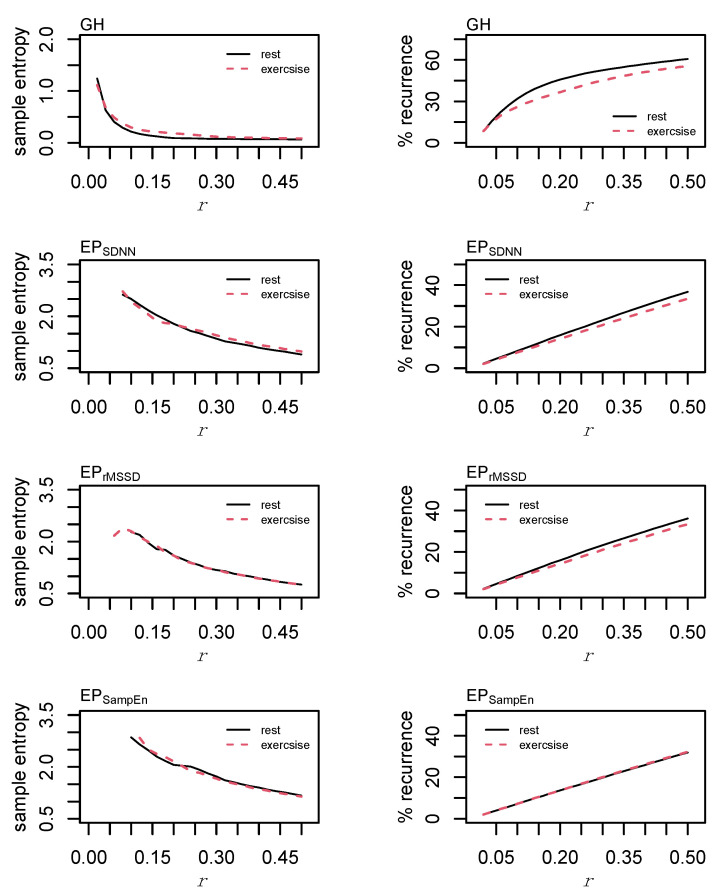
Sample entropy & recurrence analysis (RQA) optimization. Sample entropy was calculated with an embedding dimension, *m* = 2, and the radius, *r*, parameter was incremented in steps of 0.02σ from 0.02σ to 0.5σ. Recurrence quantification analysis was performed using *m* = 2, a time lag, *L* = 1, and *r* was incremented in steps of 0.02 from 0.02σ to 0.5σ.

**Figure A4:**
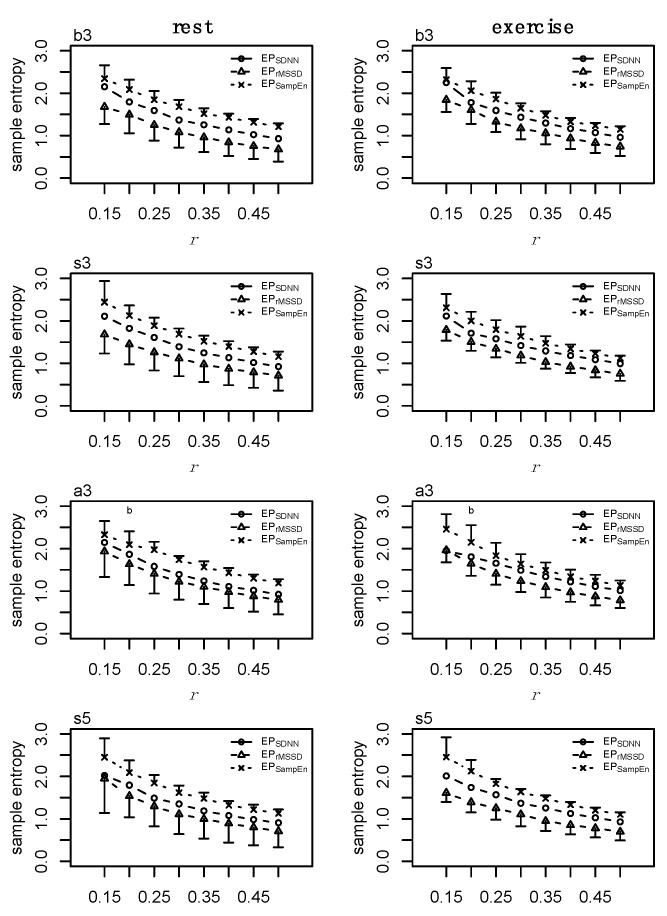
Sample entropy (SampEn) at *m* = 2 and *r* = [0.15, 0.2, 0.25, 0.3, 0.35, 0.4, 0.45, 0.5]σ for each epoched HRV profile (i.e., EP_SDNN_, EP_rMSSD_, EP_SampEn_) across each epoching method (i.e., before-3, b3; split-3, s3; after-3, a3; and split-5, s5) during rest (**first column**) and exercise (**second column**) conditions. Data presented as mean ±SD.

The individual dynamics and behaviors of GH and each of the epoched HRV profiles were examined using sample entropy and recurrence quantification analysis (RQA). For sample entropy, *m* = 2 and *r* = [0.02, 0.04, 0.06, …, 0.50]σ were examined prior to making a final decision to use *m* = 2 and *r* = 0.20σ in the formal analyses. Changes in sample entropy with respect to increases in the radius are provided in Figure 6. Sample entropy calculations for each of the epoched HRV processing methods between rest and exercise are provided in Appendix A (Figure A4).

Values of *m* = 2 and *r* = [0.02, 0.04, 0.06, …, 0.50]σ were investigated for RQA. For GH, final parameters of *m* = 2, *L* = 2, *r* = 0.04σ were chosen. Parameters were standardized across each of the epoched HRV profiles with values of *m* = 2, *L* = 1, *r* = 0.20σ. These parameters were chosen following visual inspection for a sparse matrix and comparing percent recurrence values from each of the examined radii [51]. Parameters were standardized across each of the epoched HRV profiles to permit a direct comparison between these time series. From the recurrence plots, recurrence (%REC), determinism (%DET), the average line length (LL), the total number of lines (NRLINE), laminarity (the proportion of points forming vertical lines; LAM), trapping time (TT), and Shannon entropy (ENTR) were calculated.

### 2.11. Coupling

Cross sample entropy (cross-SampEn) and cross recurrence quantification analysis (cRQA) were used to examine cardio hypothalamic-pituitary coupling. Cross sample entropy was calculated using a custom script and cRQA was performed using the “Cross-recurrence quantification analysis of categorical and continuous time series” package [52]. Specific pairings of GH-EP_SDNN_, GH-EP_rMSSD_, and GH-EP_SampEn_ were examined. A key consideration with these analyses is that these data overlay appropriately as any shift can remove the recurrences. To account for the 20-to-40-fold increase in GH concentration in response to exercise relative to the half-fold change in the epoched HRV measures, GH was log transformed and inverted. All data were subsequently mean-centered and scaled to a standard deviation of one for cross-SampEn and cRQA.

**Figure 7:**
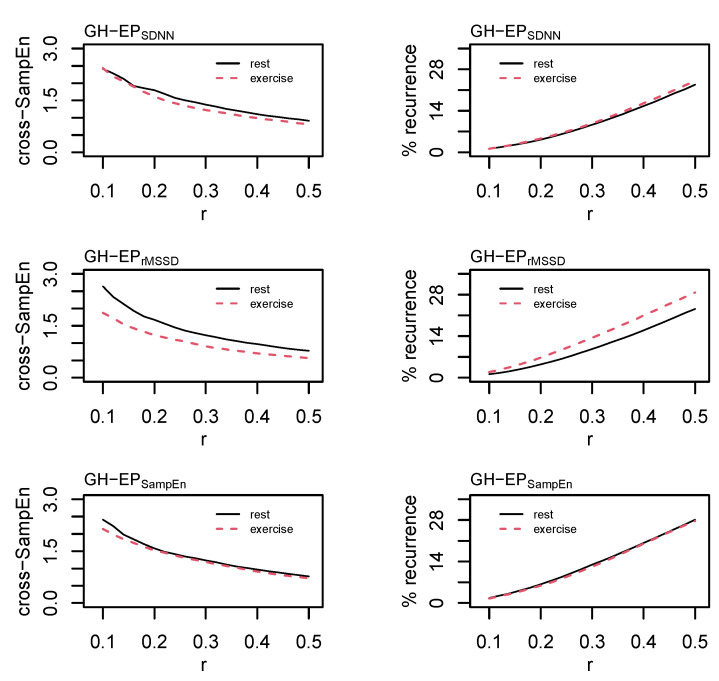
Cross sample entropy (cross-SampEn) and cross recurrence analysis (cRQA) between growth hormone (GH) and each of the epoched HRV profiles (i.e., EP_SDNN_, EP_rMSSD_, and EP_SampEn_). Cross-SampEn was calculated with an embedding dimension, *m* = 2, and the radius, *r*, parameter was incremented in steps of 0.02 from 0.02σ to 0.5σ. Cross-recurrence quantification analysis was performed using *m* = 2, a time lag, *L* = 1, and *r* was incremented in steps of 0.02 from 0.02σ to 0.5σ.

The time-delay was determined from the cross-correlation function and set at *L* = 1 for each of the three pairings. Cross-SampEn is an adaptation to the sample entropy statistic [53] and provides a measure of coupling between two time series. Values of *m* = 2 and *r* = [0.10, 0.12, 0.14, …, 0.50] were examined and the effect of an increasing tolerance/radius is provided in Figure 7. Based on these data and consideration of the total length of these time series, values of *m* = 2 and *r* = 0.20σ were chosen for the formal analysis.

Cross recurrence quantification analysis permits the visualization and quantification of the patterns between two time series without making assumptions about their statistical structure [54] and provides a highly sensitive method of assessing coupled oscillators in biology and physiology [55]. Values of *m* = 2, *L* = 1, and *r* = [0.10, 0.12, 0.14, …, 0.50]σ were examined before selecting values of *m* = 2, *L* = 1, and *r* = 0.20σ for the formal statistical analysis. As with RQA, the radius for cRQA was chosen following visual inspection for a sparse matrix and comparisons between percent recurrence values [51]. Parameters were standardized across all cRQA analyses to provide a direct comparison between GH and each of the epoched HRV profiles. Precent recurrence (%REC), %DET, LL, max line length (LL_max_), NRLINE, LAM, TT, and ENTR were calculated and compared for cRQA.

Percent recurrence (%REC) is the percentage of recurrent points within a recurrence plot and is often used as a measure to determine the optimal parameters for analysis. Percent determinism (%DET) is the proportion of recurrent points on diagonal lines and represents when a system, or two systems, occupy the same state space. In cRQA, a higher %DET value suggests a higher degree of coupling. Diagonal lines on the RQA and cRQA plots represent parallel paths/trajectories of a system, or between two systems. The total number of lines (NRLINE) provides a quantitative assessment of how often a system, or any two systems, occupy the same state space. Thus, the average line length (LL) indicates, on average, how long a system, or two systems, spend along similar paths within the reconstructed state space. Within cRQA, a higher average line length (LL) suggests a higher degree of coupling between two systems. Laminarity (LAM) is the percentage of points forming vertical (or horizontal) lines and represents a trapping of the system. Trapping time (TT) is defined as the average length of vertical lines and represents how many sequential data points/observations come back to a previously observed location. Shannon entropy from the RQA and cRQA plots is based on the distribution of the diagonal line lengths, determining probability that the length of a line will be repeated. Lower values represent a lower probability of observing more patterns while higher values represent a greater number of possible combinations and provide context to the total amount of information embedded within the plot.

### 2.12. Statistics

Paired samples t-tests were used to test for differences in body mass, body fat, fat mass, and maximal oxygen uptake, as well as measures of GH output and 24 h HRV between rest and exercise conditions. Significance was set at *p* ≤ 0.05 for all tests. All analyses were performed using R Statistics version 4.0.4 [56].

*Analysis of epoching methods*. The Hurst exponents calculated on the original, shuffle surrogate, and Gaussian surrogate data from each of the epoched HRV processing methods (i.e., b3, s3, a3, s5) were visually inspected and compared. A within subjects analysis of variance (ANOVA) was used to test for differences in the sample entropy of each of the epoched HRV profiles (i.e., EP_SDNN_, EP_rMSSD_, and EP_SampEn_) across processing methods and between conditions. The primary objective of this comparison was to determine whether there were statistically significant differences between each of the processing methods (i.e., b3, s3, a3, s5). Although this test does not determine statistical similarity, these findings were used to help determine which of the processing methods would be used for subsequent analyses.

*Dynamics of and between profiles*. Paired samples t-tests were used to test for differences in the sample entropy and RQA of GH between rest and exercise conditions. A within subjects ANOVA was used to test for differences in sample entropy between epoched HRV profiles (i.e., EP_SDNN_, EP_rMSSD_, and EP_SampEn_) and conditions (i.e., rest and exercise). Follow up comparisons were performed using Tukey’s Honest Significant Difference test.

*Cross-dynamics*. Statistical comparisons for cross-SampEn and each of the measures from cRQA were also performed using within subjects ANOVA, examining differences between crossed-profiles (i.e., GH-EP_SDNN_, GH-EP_rMSSD_, and GH-EP_SampEn_) and condition. Follow up comparisons were performed using Tukey’s Honest Significant Difference test.

*Missingness*. An average of 1% of GH samples were missing per profile and a maximum of 2.8% of samples were missing in a single subject. Growth hormone output was upsampled by a factor of 3 through cubic regression splines and the interpolated value most closely associated with the missing value[s] (in time) were used for replacement. The beginning and end of each time series were padded with three values equal to the median GH concentration of each profile to reduce overfitting at the tails. A mean of 0.2% of the epoched HRV data was missing and the largest amount of missingness associated with a single subject was 1%. Missing data associated with the epoched HRV profiles were replaced using random sampling from the observed values within each profile. This method does not retain any complex or nonlinear structure within these data; however, this was determined to be a more conservative approach to handling missingness for the purposes of these analyses.

## 3. Results

Subject characteristics during rest and exercise conditions are shown in Table 1. Consistent with previous literature [57,58], we observed a transiently higher, though non significant, total GH output over the course of the 24 h period following a single bout of aerobic exercise (t_(6)_ = l.83, *p* = 0.12), an increase in daytime GH output in response to exercise (daytime, t_(6)_ = 3.72, *p* < 0.01; exercise hours, t_(6)_ = 3.64, *p* < 0.01), and no significant differences in nighttime GH output (t_(6)_ = 0.66, *p* = 0.54). It should be noted that others have observed significant increases in 24 h GH output from both continuous and intermittent aerobic exercise [59], although, these exercise stimuli were also longer in duration than what was utilized in this study. 

The estimated Hurst exponent from the original, shuffle surrogate, and Gaussian surrogate data are presented in Table 2. The Hurst exponent can range from values between [0, 1] with values of 0.5 representing white noise. The original data for GH, as well as each of the epoched HRV profiles, have an estimated Hurst exponent >0.5 with the shuffle and Gaussian surrogate data resulting in values ≈0.5, indicating that each of the original time series contains complex or nonlinear structure.

Differences in sample entropy for each of these processing methods, rest and exercise conditions, and epoched HRV profiles were examined. No effect for the processing method was observed (F_(3, 160)_ = 1.02, *p* = 0.38) and the after-3 (i.e., a3) processing method was subsequently chosen for the formal comparisons between conditions and profiles. All models were re-run with only the a3 processing method. Table 3 provides the estimates of sample entropy and each of the measures from RQA for GH, EP_SDNN_, EP_rMSSD_, and EP_SampEn_. Sample entropy (t_(6)_ = 2.8, *p* = 0.03) of GH was increased during the exercise condition compared to rest.

**Figure 8:**
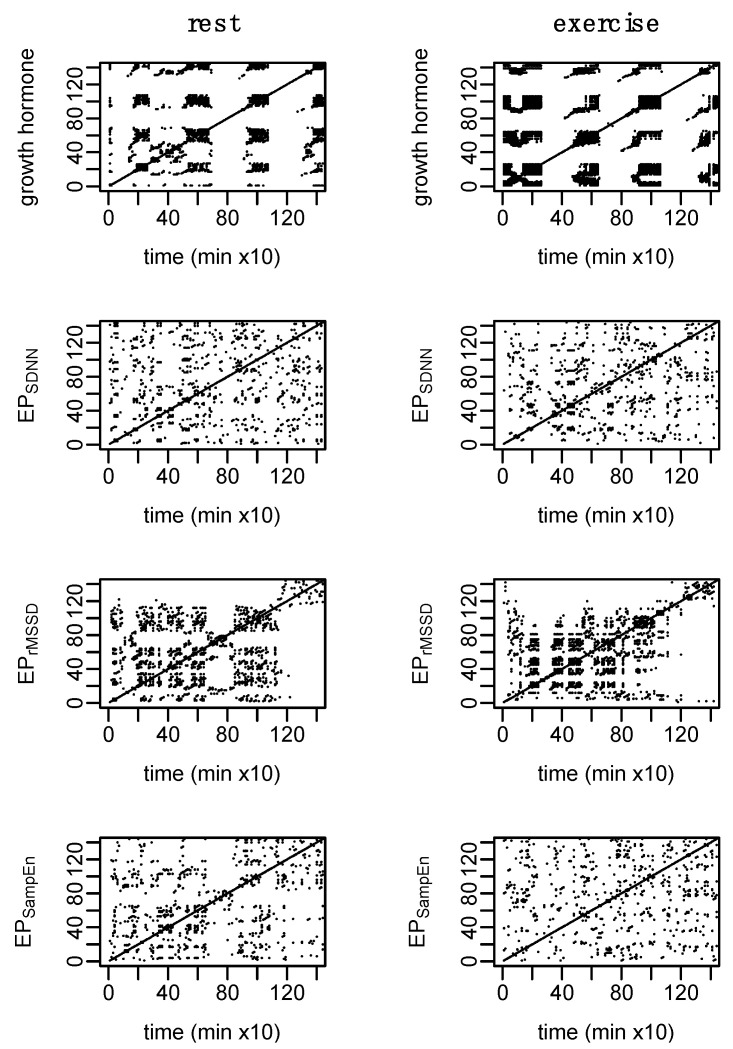
Example of recurrence analysis (RQA) for growth hormone (GH) and each of the epoched HRV profiles (i.e., EP_SDNN_, EP_rMSSD_, and EP_SampEn_). Parameters: GH, *m* = 2, *L* = 2, and *r* = 0.04σ; epoched HRV, *m* = 2, *L* = 1, and *r* = 0.20σ. Consistent with previous figures, these data are from subject 3.

While there are some qualitative differences observed in the RQA plots (Figure 8), none of the measures from RQA were different between conditions: %REC (t_(6)_ = 0.6, *p* = 0.60), %DET (t_(6)_ = 0.2, *p* = 0.85), NRLINE (t_(6)_ = 1.5, *p* = 0.17), LL (t_(6)_ = 1.8, *p* = 0.13), LAM (t_(6)_ = 0.3, *p* = 0.80), TT (t_(6)_ = 1.4, *p* = 0.21), ENTR (t_(6)_ = 0.8, *p* = 0.43). These *p*-values have not been adjusted for multiple comparisons which may increase the family-wise error rate.

Parameters for sample entropy and RQA were standardized across the epoched HRV profiles, providing a direct comparison in the regularity and dynamic behaviors of these profiles relative to the others. Main effects for profile were observed for sample entropy (F_(2, 30)_ = 9.8, *p* < 0.001), %REC (F_(2, 30)_ = 4.8, *p* = 0.02), %DET (F_(2, 30)_ = 11.5, *p* < 0.001), NRLINE (F_(2, 30)_ = 9.0, *p* < 0.001), LL (F_(2, 30)_ = 4.7, *p* = 0.02), LAM (F_(2, 30)_ = 11.3, *p* < 0.001), TT (F_(2, 30)_ = 5.9, *p* < 0.01), and ENTR (F_(2, 30)_ = 12.4, *p* < 0.001). Additionally, a main effect for condition was observed within the analysis of %REC (F_(1, 30)_ = 6.3, *p* < 0.05). Pairwise comparisons are provided in Table 3. Figure 8 provides an example of the RQA plots for a single subject.

**Figure 9:**
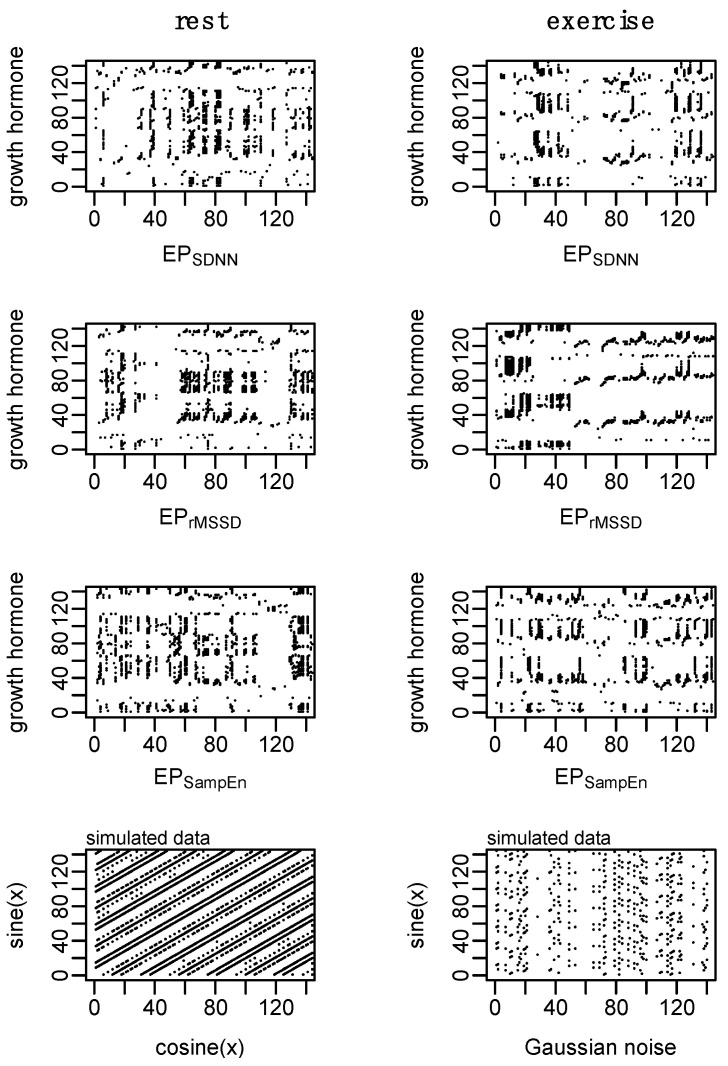
Example of cross recurrence analysis (cRQA) for growth hormone (GH) and each of the epoched HRV profiles (i.e., GH-EP_SDNN_, GH-EP_rMSSD_, and GH-EP_SampEn_) to assess cardio hypothalamic-pituitary coupling. Simulated data aim to provide visual context of two coupled systems and a highly deterministic system coupled with random noise. Parameters: *m* = 2, *L* = 1, and *r* = 0.20. Consistent with previous figures, these data are from subject 3.

Cardio hypothalamic-pituitary coupling was assessed through the pairings of GH with each of the epoched HRV profiles (i.e., GH-EP_SDNN_, GH-EP_rMSSD_, and GH-EP_SampEn_). Results from the analysis of cross-SampEn and cRQA are provided in Table 4 and an example of the cRQA plots for a single subject is provided in Figure 9. An interaction between profile and condition was observed for cross-SampEn (F_(2, 30)_ = 3.5, *p* = 0.04); pairwise comparisons are provided in Table 4. 

In addition to some qualitative differences between coupled profiles observed in the cRQA plots, several main effects and interactions were observed from the analysis of quantitative features of cRQA: %REC (interaction, F_(2, 30)_ = 8.1, *p* < 0.01); %DET (interaction, F_(2, 30)_ = 3.6, *p* = 0.04); NRLINE (interaction, F_(2, 30)_ = 8.2, *p* < 0.01); LL_max_ (condition, F_(1, 30)_ = 5.7, *p* = 0.02; profile, F_(2, 30)_ = 10.4, *p* < 0.001); LL (condition, F_(1, 30)_ = 4.5, *p* = 0.04; profile, F_(2, 30)_ = 7.6, *p* < 0.01), LAM (condition, F_(1, 30)_ = 9.6, *p* < 0.01; profile F_(2, 30)_ = 5.3, *p* = 0.01), TT (condition, F_(1, 30)_ = 4.4, *p* = 0.04; profile, F_(2, 30)_ = 5.9, *p* < 0.01); and ENTR (condition, F_(1, 30)_ = 5.7, *p* = 0.02; profile F_(2, 30)_ = 8.4, *p* < 0.01).

## 4. Discussion

The objective of this study was to examine cardio hypothalamic-pituitary coupling and to better understand how the temporal relations between these systems are altered during rest and exercise conditions. To address issues of timescale invariance between GH and HRV, we first examined methods of processing a continuous RR-recording into discrete short-time assessments of HRV (epoched HRV) to explore the physiological meaning of these time series. We subsequently examined the time-dependent and temporal relations between these measures of cardiac dynamics with growth hormone. In general, our hypotheses were supported as we did observe coupling between the epoched HRV profiles and GH during rest and exercise. However, the coupling between the epoched HRV profiles and GH was differentially impacted by the exercise stimulus which does have practical implications for future work and future directions.

The effects of exercise on GH output are well established and the present findings are consistent with previous literature [57,58]. In addition to measures of GH output, previous works have also examined the effects of exercise, aging, pharmacological intervention, and disease, on the regularity of GH output [31,58]. The effects of the high-intensity exercise bout on GH output can clearly be observed in Figure 2 (top right) and the corresponding state space reconstruction can be observed in Figure 4. The notable, but non significant, increase in 24 h GH output following exercise was coupled with an increase in the irregularity of GH output, indicating altered hypothalamic-pituitary dynamics.

Heart rate variability of 24 h RR-recordings are a gold standard in clinical settings and have been linked to several cardiovascular and cardiometabolic diseases [24,25]. Throughout the 24 h period, both parasympathetic and sympathetic inputs contribute to changes in SDNN, while rMSSD primarily represents vagally mediated inputs [25]. In the present study, 24 h SDNN was elevated during the exercise condition while 24 h rMSSD was unchanged, suggesting that vagally mediated inputs (across the entire 24 h period) were not impacted by the exercise stimulus and that the observed changes in the 24 h SDNN measures are attributable to altered sympathetic and neurohumoral inputs; consistent with what would be expected from a high-intensity exercise bout. We also observed a significant reduction in the sample entropy of the 24 h RR-recording with exercise, representing a more regular pattern within the RR-intervals.

Generally, the loss of variability and complexity within a system represents a reduced capacity of that system to respond to a perturbation [19,20,21,22] and chronic adaptations, such as the manifestation of disease, have been shown to alter the dynamics between subsystems [27,28,29,60]. In more dynamic environments, such as those that may include an exercise bout, changes in the variability or complexity of a time series may be the result of the perturbation itself and not a robust representation of the adaptability of the system. Such biases may be particularly relevant depending on the scale of the data, the underlying trends, and how these trends are handled within the analysis [61].

### 4.1. Heart Rate Variability

In addition to 24 h measurements of SDNN, rMSSD, and SampEn, we examined the dynamics of autonomic control on the heart throughout the 24 h period through serial short-time measurements of HRV. Short-time HRV measurements provide an assessment of cardiac autonomic control within a specific window and provide different information about the overall status of the system compared to a 24 h measurement [25]. These short-time HRV measurements are driven by changes in autonomic control, respiratory sinus arrhythmia, and the baroreceptor-reflex [25]. As such, these serially assessed short-time HRV measurements (i.e., epoched HRV) provided information regarding changes in acute regulatory inputs to cardiac dynamics throughout the course of a 24 h period compared to an average across the entire 24 h period. Previously, serial measurement of short-time HRV has been used to examine diurnal patterns of cardiac autonomic regulation between genders and across age groups [62], as well as to investigate compounding physical stress on the autonomic nervous system [63]. However, to the best of our knowledge, there have been no prior works examining the dynamics of these serial short-time HRV measurements (i.e., epoched HRV).

We assessed four separate processing methods to determine the robustness of epoched HRV at varying sampling periods and window lengths. Regardless of the method, each epoch was initiated on a 10 min increment (from start to start). A variety of timing intervals (e.g., 15, 20, or 30-min) could have been chosen but these 10 min intervals were selected to coincide with the GH sampling. Shuffle and Gaussian surrogate data were generated to examine whether these epoched profiles differed from random noise. The Hurst exponent from the raw data ranged from ~0.6 to ~0.7 and fell to values of ~0.5 for each of the surrogate data, highlighting the existence of a nonlinear or complex structure. Epoched HRV data (Figure 2) and the state space reconstructions of these data (Figure 4 and Figure A3) provide some visual context to these observations. While there are some qualitative signs within the state space reconstruction of the epoched HRV profiles, the stochastic nature of these data creates noisy visuals and as a result, we have relied heavily on the entirety of these analyses to support the existence of nonlinear structure within these epoched HRV profiles.

With four processing methods to choose from, we first examined whether there were differences in the sample entropy of each of these processing methods. While this did not establish statistical similarity, it did indicate that the regularity of each of these time series was not different across processing methods and as a result, subsequent analyses were performed using the after-3 (a3) processing method. In contrast to the increase in SDNN and decrease in SampEn observed in the 24 h measurements, exercise did not alter the sample entropy of the epoched HRV profiles. However, a higher degree of regularity (lower sample entropy) in EP_SDNN_ and EP_rMSSD_ compared to EP_SampEn_ was observed, suggesting that these epoched profiles likely represent different information regarding the physiologic regulatory inputs on the heart compared to the 24 h measurements. More specifically, these serially assessed short-time measurements of SDNN and rMSSD (i.e., EP_SDNN_ and EP_rMSSD_) reflect acute changes in cardiac control related to autonomic control, respiratory sinus arrhythmia, and the baroreceptor-reflex while EP_SampEn_ provides an assessment of acute changes in cardiac complexity across the 24 h period. Thus, the sample entropy statistic calculated on the epoched HRV profiles represents a measure of regularity within changes of short-time measurements of cardiac control and regularity (i.e., regularity of short-time regularity) whereas the 24 h measurements represent a global shift across the entire period.

Visually, there are clear differences in the recurrent patterns of EP_SDNN_, EP_rMSSD_, and EP_SampEn_ (example provided in Figure 8). One of the largest visual differences observed between the epoched HRV profiles can be seen in the transition into the nighttime hours (particularly during rest and especially for EP_rMSSD_). This shift represents a trend within the data, which can also be observed in Figure 2, and as is commonly observed in raw 24 h RR recordings. During the exercise condition, this nighttime trend is reduced. Quantitatively, the total number of lines, and laminarity (percent of the plot made up of vertical and/horizontal lines), were lower for EP_SampEn_ compared to both EP_SDNN_ and EP_rMSSD_. This difference in the total number of points indicates fewer recurrent points for the EP_SampEn_ profiles while the reduced laminarity suggests that the EP_SampEn_ profiles spent less time trapped within the same state space. These quantitative measures from the RQA plots provide important information regarding the dynamics of each system, as well as a comparison between systems. However, despite the lack of statistical significance in these differences, the visual interpretation of these plots suggests important information is represented by these differences and thus, their interpretation should not be underestimated.

### 4.2. Cardio-Hypothalamic-Pituitary Coupling

Interdependence, or coupling, between physiologic systems is an interesting and important phenomenon with clear importance in physiology. There are two primary categories of coupling, including the instance where one system directly regulates the other, and generalized coupling where two systems progress through time and state space with similar responses to internal and external perturbations. While there are no singular measures to determine the coupling between systems, there are a variety of tools that can help provide insight. When dealing with deterministic systems, being able to forecast the values of one system from another would suggest a functional relationship between them. In some cases, these predictions may be reciprocal while in other instances, prediction performance may be unidirectional. In either case, this would provide evidence of coupling between these two systems. Alternatively, topological mapping between the systems would also suggest that the attractors of these systems are connected through a nonlinear function.

Due to the stochastic nature of the epoched HRV profiles, we have not taken on the task of mutual forecasting to establish interdependence and instead, our efforts have focused on the use and application of cross-SampEn and cRQA. Both cross-SampEn and cRQA are proven and useful tools to examine the relations between one or two stochastic signals. Just as we would interpret the sample entropy statistic of an individual time series, higher values of cross-SampEn represent more irregularity within the two signals or less coupling between the two systems. Within the cRQA plots, each recurrence (dot on the cRQA plot) represents when the two systems occupy similar spaces in the state space. Thus, each line represents an occasion where the trajectory of these two systems is close in proximity and the line lengths represent the duration that these systems share that trajectory. To examine cardio hypothalamic-pituitary coupling, we have assessed the relations between GH and each of the epoched HRV profiles (i.e., GH-EP_SDNN_, GH-EP_rMSSD_, and GH-EP_SampEn_).

Overall, the cross-SampEn of GH-EP_SDNN_, GH-EP_rMSSD_, and GH-EP_SampEn_ were differentially affected by the condition. During rest, the cross-SampEn of these three pairings were not different, indicating a similar degree of regularity in the coupling of these systems. However, the cross-SampEn of GH-EP_rMSSD_ was reduced during the exercise condition and notably, the reduction in cross-SampEn of GH-EP_SDNN_ during the exercise condition approached significance. In both cases, this suggests that exercise altered the relations between GH and each of the epoched HRV profiles (i.e., EP_SDNN_ and EP_rMSSD_) by establishing tighter coupling between the two systems. Quantitative analysis from cRQA supported these findings, though, the statistical analysis only indicated significant changes for GH-EP_rMSSD_ and not GH-EP_SDNN_. For GH-EP_rMSSD_, exercise resulted in a larger number of recurrences (NRLINE) and an increase in the maximal time spent along similar trajectories (LL_max_), leading to an increase in the overall determinism between the two systems. This increase in determinism following the exercise condition was coupled with an increase in laminarity (% of recurrences forming vertical or horizontal lines). While trapping time (TT-average length of vertical or horizontal lines) was not different between conditions, trapping time for GH-EP_rMSSD_ was higher than both GH-EP_SDNN_ and GH-EP_SampEn_.

Shannon entropy of cRQA plots is based on the distribution of diagonal line lengths and establishes the probability that any given line length will be repeated. Thus, the elevated Shannon entropy in GH-EP_rMSSD_ suggests a higher number of possible patterns shared between the two systems compared to both GH-EP_SDNN_ and GH-EP_SampEn_. This can be loosely visualized in Figure 9 where the density and types of line structures in the GH-EP_rMSSD_ plot are somewhat different from those observed in the plots of GH-EP_SDNN_ and GH-EP_SampEn_.

Short-time measures of rMSSD are more heavily influenced by the parasympathetic nervous system and predominantly represent changes in vagally mediated inputs [25], while short-time measures of SDNN are not only influenced by sympathetic and parasympathetic inputs, but are also dependent on other measurement conditions and the impact of parasympathetically mediated respiratory sinus arrhythmia [25]. As such, the pairing of GH-EP_rMSSD_ provides a representation of the coupling between GH and vagally mediated inputs while GH-EP_SDNN_ provides a more global representation of GH and inputs on cardiac control. Further, the increased coupling in GH-EP_rMSSD_ during the exercise condition, compared to the non significant increase in coupling between GH-EP_SDNN_, emphasize the potential application for each of these measures in different circumstances. Qualitatively, there is a clear shift in the coupling between GH-EP_rMSSD_ during the exercise condition with afternoon and nighttime measurements of rMSSD occupying a similar state space as the GH response immediately following exercise (Figure 9, second row). This pattern is, somewhat, observed with GH-EP_SDNN_ (Figure 9, first row) but not with GH-EP_SampEn_ (Figure 9, third row).

The dynamics of GH-EP_SampEn_ were not different between rest and exercise conditions, nor did the cross-SampEn or quantitative measures from cRQA trend in that direction. Interestingly, compared to both GH-EP_SDNN_ and GH-EP_rMSSD_, the pairing of GH-EP_SampEn_ contained a larger number of overall recurrences (NRLINE) during the rest condition. However, the exercise condition resulted in an increase in the number of lines for both GH-EP_SDNN_ and GH-EP_rMSSD_. For GH-EP_SDNN_, the total number of lines became more alike to GH-EP_SampEn_ while the total number of lines for GH-EP_rMSSD_ increased drastically and became significantly more than for GH-EP_SampEn_. The Shannon entropy of these pairings followed a similar trend, with that of GH-EP_rMSSD_ being higher than both GH-EP_SDNN_ and GH-EP_SampEn_ while the observed increase in Shannon entropy of GH-EP_SampEn_ was not different from that of GH-EP_SDNN_.

Overall, these findings provide interesting insights regarding cardio hypothalamic-pituitary coupling and how any of these epoched HRV profiles might be used to examine these phenomena. However, these findings also pose interesting questions regarding the underlying regulatory dynamics of these systems—primarily those of the epoched HRV profiles. The EP_SDNN_ profiles, in theory, represent a more global measure of acute changes in parasympathetic, sympathetic, respiratory, and other humoral inputs while the EP_rMSSD_ profiles primarily represent vagally mediated inputs. Conversely, the EP_SampEn_ profiles represent acute changes in cardiac dynamics, representative of more complex regulatory dynamics and indicative of the overall adaptability of the system. Further assessing the dynamics of these time series with GH through cross-SampEn and cRQA provides a means of assessing how changes in cardiac control similarly, or differentially, couple with hypothalamic-pituitary regulation.

The reduction in cross-SampEn and overall increase in the determinism of GH-EP_rMSSD_ in response to exercise suggests that the EP_rMSSD_ profile may not provide a robust means to examine cardio hypothalamic-pituitary coupling. More specifically, although the coupling between GH and short-time assessments of [predominantly] vagally mediated inputs increased during the exercise condition, this also suggests that the dynamics of vagally mediated inputs on cardiac control (i.e., through EP_rMSSD_) are not associated with hypothalamic-pituitary regulation at rest. Although measures from cRQA were not different for GH-EP_SDNN_, several of these measures trended higher during the exercise condition. These observations were coupled with a noticeable reduction in cross-SampEn (of GH-EP_SDNN_) during the exercise condition, further suggesting that cardiac dynamics, assessed through EP_rMSSD_, are more tightly coupled with hypothalamic-pituitary regulation following exercise compared to rest. The percent recurrence and percent determinism were similar across all pairings of GH and the epoched HRV profiles. However, a higher total number of recurrent points for GH-EP_SampEn_ during the rest condition (compared to GH-EP_SDNN_ and GH-EP_rMSSD_) and a similar number of recurrent points during the exercise condition, suggest that the EP_SampEn_ profile may be more tightly coupled with hypothalamic-pituitary regulation compared to EP_SDNN_ and EP_rMSSD_.

### 4.3. Limitations

The current sample includes a homogenous sample of seven subjects and although we utilized a robust within subject design, the generalizability of these findings is limited, and future works should examine the reproducibility of these data, as well as explore cardio hypothalamic-pituitary coupling in other healthy and diseased populations. Additionally, future works should consider longer periods of sampling. The false nearest neighbors algorithm used to estimate the proper embedding dimension has been shown to be affected by noise [45,46] and data length [47]. Thus, these longer sampling periods would permit additional power in estimating the embedding dimension.

A variety of factors, including age, body fat, fat-free mass, and maximal oxygen uptake are known to impact GH output and secretory dynamics [59,64,65,66,67]. Similarly, HRV has been shown to change across the lifespan and with the manifestation of disease [24,25]. As part of the effort to understand how the shared dynamics between the hypothalamic-pituitary axis and cardiac control might differ in response to various perturbations, as well as chronic adaptations, these factors should be considered and explored in future works with a more diverse sample. Additionally, future works targeting specific mechanisms that either up-regulate or inhibit GH and/or cardiac autonomic control through pharmacological intervention may reveal important information regarding cardio hypothalamic-pituitary coupling. Further, the assessment of biomarkers that have dual-regulatory roles between cardiac control and hypothalamic-pituitary regulation may provide context to the temporal organization and regulation between these systems. Potential markers might include galanin and/or nucleobindin-2/nesfatin-1. Similar to GH and HRV, both galanin [68,69,70,71,72,73] and nesfatin-1 [74,75,76,77] are altered with disease and have been previously linked to hypothalamic-pituitary-adrenal axis functioning [70,71,78,79,80].

## 5. Concluding Remarks and Future Directions

The study of cardio hypothalamic-pituitary coupling poses several challenges with specific issues related to timescale invariance. The use of epoched HRV provides a method of examining the temporal changes in cardiac control with hypothalamic-pituitary regulation using short-time measures of HRV. We have outlined some key differences in the physiological interpretation of these data relative to more traditional 24 h measurements and subsequently examined how these time series couple with GH output during rest and exercise conditions. These epoched HRV profiles are not necessarily better, or worse, than the 24 h measurement—merely different—and may provide opportunities for technological and analytical developments in exercise, sport, military, and clinical settings.

The coupling between each of these epoched HRV profiles with GH provides context to the dynamics between the cardiac and hypothalamic-pituitary regulation. Increases in the coupling between EP_SDNN_ and EP_rMSSD_ with GH during the exercise condition highlight the association between the autonomic nervous system with cardiac and hypothalamic-pituitary regulation. For the purposes of examining dynamics associated with autonomic function, the EP_SDNN_ and EP_rMSSD_ profiles may provide a means of understanding how cardio hypothalamic-pituitary coupling is altered through the manifestation of cardiovascular and cardiometabolic diseases. Conversely, GH-EP_SampEn_ coupling appears relatively robust to the exercise stimulus and may provide a method for integrating these data into other analytical frameworks. One example may be the estimation of underlying hypothalamic-pituitary regulatory dynamics through the noninvasive assessment of cardiac dynamics. Nevertheless, as future work builds on these findings and potential applications are contemplated, such considerations should be carefully paired with project objectives.

## Figures and Tables

**Table 1:** Subject demographics, growth hormone (GH) output, and 24 h measures of heart rate variability (HRV) during rest and exercise conditions.

		Rest	Exercise
	Age (years)	25.4 (±2.6)	-
	Height (cm)	174.7 (±7.8)	-
	Body mass (kg)	72.5 (±13.7)	74.3 (±13.2)
	Body fat (%)	9.46 (±2.88)	10.59 (±3.8)
	Fat mass (kg)	6.98 (±2.7)	8.05 (±3.8)
	VO_2max_ (mL/kg/min)	66.9 (±8.7)	70.1 (±10.8)
GH	Total (24 h) (ng)	1083.3 (±152.5)	1596.7 (±276.9)
	Daytime (ng)	307.0 (±77.5) *	735.1 (±108.1)
	Nighttime (ng)	679.4 (±85.8)	785.9 (±166.9)
	Exercise (ng)	73.1 (±39.4) *	458.1 (±91.2)
	Nighttime peak (ng/mL)	5.5 (±0.9)	5.5 (±1.4)
	Exercise peak (ng/mL)	0.8 (±0.4) *	7.8 (±1.6)
	Nadir (ng/mL)	0.1 (±0.03)	0.1 (±0.03)
24 h HRV	SDNN	181.5 (±49.4) *	210.9 (±42.6)
	rMSSD	76.2 (±35.3)	75.9 (±36.8)
	SampEn	1.61 (±0.22) *	0.75 (±0.07)

* *p* < 0.05. Demographic information is presented as mean (±SD); GH data are presented as mean (±SEM); HRV data are presented as mean (±SD). Maximal oxygen uptake (VO_2max_); standard deviation of the normal RR-interval (SDNN); the root mean square of successive differences (rMSSD); sample entropy (SampEn).

**Table 2:** Hurst exponents for the original, shuffle surrogate, and gaussian surrogate data for growth hormone (GH) and each of the epoched HRV profiles (i.e., EP_SDNN_, EP_rMSSD_, and EP_SampEn_) during the rest and exercise conditions.

			Rest			Exercise	
	Method	Observed	Shuffle	Gaussian	Observed	Shuffle	Gaussian
GH	-	0.73 (±0.03)	0.50 (±0.05)	0.51 (±0.06)	0.68 (±0.03)	0.53 (±0.05)	0.53 (±0.04)
EP_SDNN_	b3	0.68 (±0.08)	0.52 (±0.05)	0.53 (±0.06)	0.68 (±0.06)	0.48 (±0.03)	0.53 (±0.06)
	s3	0.65 (±0.09)	0.52 (±0.04)	0.53 (±0.04)	0.65 (±0.07)	0.52 (±0.05)	0.52 (±0.06)
	a3	0.66 (±0.07)	0.53 (±0.06)	0.51 (±0.04)	0.65 (±0.06)	0.52 (±0.05)	0.52 (±0.07)
	s5	0.67 (±0.09)	0.52 (±0.04)	0.52 (±0.05)	0.67 (±0.06)	0.54 (±0.04)	0.51 (±0.04)
EP_rMSSD_	b3	0.71 (±0.09)	0.52 (±0.04)	0.51 (±0.05)	0.74 (±0.05)	0.52 (±0.04)	0.53 (±0.03)
	s3	0.71 (±0.1)	0.52 (±0.05)	0.52 (±0.06)	0.73 (±0.05)	0.49 (±0.04)	0.53 (±0.03)
	a3	0.71 (±0.09)	0.52 (±0.06)	0.53 (±0.06)	0.73 (±0.04)	0.51 (±0.04)	0.51 (±0.05)
	s5	0.72 (±0.09)	0.49 (±0.04)	0.55 (±0.04)	0.74 (±0.05)	0.52 (±0.05)	0.53 (±0.06)
EP_SampEn_	b3	0.60 (±0.06)	0.57 (±0.02)	0.51 (±0.05)	0.64 (±0.05)	0.54 (±0.05)	0.52 (±0.03)
	s3	0.59 (±0.08)	0.50 (±0.04)	0.52 (±0.04)	0.62 (±0.05)	0.54 (±0.03)	0.53 (±0.05)
	a3	0.61 (±0.08)	0.54 (±0.03)	0.52 (±0.04)	0.63 (±0.05)	0.53 (±0.04)	0.51 (±0.05)
	s5	0.60 (±0.08)	0.53 (±0.05)	0.48 (±0.05)	0.64 (±0.03)	0.52 (±0.06)	0.50 (±0.06)

Data presented as mean (±SD).

**Table 3:** Sample entropy (SampEn) and recurrence quantification analysis (RQA) for growth hormone (GH) and each of the epoched HRV profiles (i.e., EP_SDNN_, EP_rMSSD_, EP_SampEn_) during the rest and exercise conditions.

		Rest	Exercise
GH	SampEn	0.10 (±0.03) *	0.18 (±0.09)
	%REC	15.8 (±5.0)	14.9 (±5.5)
	%DET	64.1 (±8.7)	65.8 (±21.7)
	NRLINE	715.3 (±292.3)	554.7 (±236.6)
	LL	3.0 (±0.2)	3.9 (±1.2)
	LAM (%)	73.1 (±7.1)	70.7 (±22.6)
	TT	3.3 (±0.5)	4.6 (±2.2)
	ENTR	1.22 (±0.18)	1.44 (±0.70)
EP_SDNN_	SampEn ^b^	1.78 (±0.20)	1.78 (±0.18)
	%REC	15.9 (±2.3) *	14.2 (±1.1)
	%DET ^a^	35.2 (±3.0)	33.2 (±2.1)
	NRLINE ^b^	468.4 (±101.6)	382.1 (±58.6)
	LL ^a^	2.5 (±0.1)	2.6 (±0.1)
	LAM (%) ^b^	44.9 (±4.1)	39.7 (±4.8)
	TT	2.5 (±0.1)	2.4 (±0.1)
	ENTR ^a^	0.57 (±0.08)	0.59 (±0.04)
EP_rMSSD_	SampEn ^c^	1.60 (±0.52)	1.60 (±0.26)
	%REC ^c^	16.0 (±2.8) *	14.3 (±0.7)
	%DET ^a,c^	41.5 (±13.8)	40.0 (±6.5)
	NRLINE ^c^	536.4 (±240.9)	456.4 (±82.2)
	LL ^a^	2.7 (±0.2)	2.7 (±0.1)
	LAM (%) ^c^	47.9 (±17.9)	49.6 (±6.9)
	TT ^c^	2.6 (±0.5)	2.5 (±0.2)
	ENTR ^a,c^	0.74 (±0.31)	0.73 (±0.14)
EP_SampEn_	SampEn ^b,c^	2.06 (±0.34)	2.16 (±0.43)
	%REC ^c^	13.6 (±0.4)	13.6 (±0.5)
	%DET ^c^	28.9 (±1.9)	29.5 (±3.2)
	NRLINE ^b,c^	317.3 (±27.3)	325.3 (±49.5)
	LL	2.6 (±0.1)	2.61 (±0.1)
	LAM (%) ^b,c^	32.7 (±6.6)	33.6 (±5.0)
	TT ^c^	2.3 (±0.1)	2.3 (±0.1)
	ENTR ^c^	0.46 (±0.08)	0.48 (±0.11)

SampEn parameters: GH, *m* = 2 and *r* = 0.20σ; epoched HRV, *m* = 2 and *r* = 0.20σ. RQA parameters: GH, *m* = 2, *L* = 2, and *r* = 0.04σ; epoched HRV, *m* = 2, *L* = 1, and *r* = 0.20σ. Pairwise comparisons (*p* < 0.05) of sample entropy ^a^ EP_SDNN_ vs. EP_rMSSD_; ^b^ EP_SDNN_ vs. EP_SampEn_; ^c^ EP_rMSSD_ vs. EP_SampEn_. Pairwise comparisons between rest and exercise conditions are denoted: *, *p* < 0.05.

**Table 4:** Cross sample entropy (cross-SampEn) and cross-recurrence analysis (cRQA) between GH and each of the epoched HRV profiles (i.e., GH-EP_SDNN_, GH-EP_rMSSD_, and GH-EP_SampEn_) during the rest and exercise conditions.

		Rest	Exercise
GH-EP_SDNN_	Cross-SampEn	1.80 (±0.25) †	1.61 (±0.22) ^a^
	%REC	4.3 (0.8) ^b^	4.6 (0.7) ^a^
	%DET	43.4 (6.0)	48.4 (6.3) ^a^
	NRLINE	169.6 (46.5) ^b^	196.6 (43.5) ^a^
	LL_Max_ ^a^	4.9 (0.9)	5.3 (1)
	LL ^a^	2.3 (0.1)	2.4 (0.1)
	LAM ^a^	35.5 (7.6)	40.7 (10.7)
	TT ^a^	2.3 (0.2)	2.4 (0.2)
	ENTR ^a^	0.66 (0.15)	0.79 (0.15)
GH-EP_rMSSD_	Cross-SampEn	1.67 (±0.27) *	1.23 (±0.25) ^a,c^
	%REC	4.5 (1.0) ^c,^*	6.8 (1.5) ^a^
	%DET	47.8 (7.5) *	59.7 (9.4) ^a,c^
	NRLINE	180.7 (55.7) ^c,^*	316.3 (82.4) ^a,c^
	LL_Max_ ^a^	6.1 (0.7) *	8.0 (1.6)
	LL ^a,c^	2.5 (0.1)	2.7 (0.3)
	LAM ^a^	39.5 (11.2) *	55.5 (10.1)
	TT ^a,c^	2.5 (0.1)	2.8 (0.4)
	ENTR ^a,c^	0.91 (0.15)	1.05 (0.25)
GH-EP_SampEn_	Cross-SampEn	1.58 (±0.20)	1.52 (±0.13) ^c^
	%REC	6.3 (1.3)	5.9 (0.5)
	%DET	47.9 (6.6)	47.9 (5.5) ^c^
	NRLINE	269.6 (88.5) ^b,c^	239.4 (40.2) ^c^
	LL_max_	6 (1.5)	6.3 (1)
	LL ^c^	2.4 (0.1)	2.4 (0.1)
	LAM	38.3 (12.5)	40.8 (8.6)
	TT ^c^	2.3 (0.2)	2.4 (0.1)
	ENTR ^c^	0.77 (0.16)	0.87 (0.1)

Cross sampEn parameters: *m* = 2 and *r* = 0.20. cRQA parameters: *m* = 2, *L* = 1, and *r* = 0.20. Pairwise comparisons (*p* < 0.05) of cross-SampEn and cRQA values between profiles are denoted: ^a^ GH-EP_SDNN_ vs. GH-EP_rMSSD_; ^b^ GH-EP_SDNN_ vs. GH-EP_SampEn_; ^c^ GH-EP_rMSSD_ vs. GH-EP_SampEn_. Pairwise comparisons between rest and exercise conditions are denoted: *, *p* < 0.05; †, *p* < 0.1.

## Data Availability

Data are available from NTB or LW upon request.

## References

[1] Peng C.K., Havlin S., Stanley H.E., Goldberger A.L. (1995). Quantification of scaling exponents and crossover phenomena in nonstationary heartbeat time series. Chaos.

[2] Peng C.K., Mietus J., Hausdorff J.M., Havlin S., Stanley H.E., Goldberger A.L. (1993). Long-range anticorrelations and non-Gaussian behavior of the heartbeat. Phys. Rev. Lett..

[3] Berry N.T., Bechke E., Shriver L.H., Calkins S.D., Keane S.P., Shanahan L., Wideman L. (2021). Heart Rate Dynamics During Acute Recovery From Maximal Aerobic Exercise in Young Adults. Front. Physiol..

[4] Chiang J.Y., Huang J.W., Lin L.Y., Chang C.H., Chu F.Y., Lin Y.H., Wu C.K., Lee J.K., Hwang J.J., Lin J.L. (2016). Detrended Fluctuation Analysis of Heart Rate Dynamics Is an Important Prognostic Factor in Patients with End-Stage Renal Disease Receiving Peritoneal Dialysis. PLoS ONE.

[5] Goldberger A.L., Amaral L.A., Hausdorff J.M., Ivanov P., Peng C.K., Stanley H.E. (2002). Fractal dynamics in physiology: Alterations with disease and aging. Proc. Natl. Acad. Sci. USA.

[6] Schulte-Frohlinde V., Ashkenazy Y., Goldberger A.L., Ivanov P., Costa M., Morley-Davies A., Stanley H.E., Glass L. (2002). Complex patterns of abnormal heartbeats. Phys. Rev. E Stat. Nonlinear Biol. Soft Matter Phys..

[7] Hausdorff J.M., Peng C.K., Ladin Z., Wei J.Y., Goldberger A.L. (1995). Is walking a random walk? Evidence for long-range correlations in stride interval of human gait. J. Appl. Physiol..

[8] Hausdorff J.M., Edelberg H.K., Mitchell S.L., Goldberger A.L., Wei J.Y. (1997). Increased gait unsteadiness in community-dwelling elderly fallers. Arch. Phys. Med. Rehabil..

[9] Hausdorff J.M. (2007). Gait dynamics, fractals and falls: Finding meaning in the stride-to-stride fluctuations of human walking. Hum. Mov. Sci..

[10] Rhea C.K., Kiefer A.W. (2014). Patterned variability in gait behavior: How can it be measured and what does it mean?. Gait Biometrics: Basic Patterns, Role of Neurological Disorders and Effects of Physical Activity.

[11] Rhea C.K., Kiefer A.W., Wittstein M.W., Leonard K.B., MacPherson R.P., Wright W.G., Haran F.J. (2014). Fractal gait patterns are retained after entrainment to a fractal stimulus. PLoS ONE.

[12] Stergiou N., Decker L.M. (2011). Human movement variability, nonlinear dynamics, and pathology: Is there a connection?. Hum. Mov. Sci..

[13] Collins S.H. (2008). Dynamic Walking Principles Applpied to Human Gait.

[14] Manor B., Costa M.D., Hu K., Newton E., Starobinets O., Kang H.G., Peng C.K., Novak V., Lipsitz L.A. (2010). Physiological complexity and system adaptability: Evidence from postural control dynamics of older adults. J. Appl. Physiol..

[15] Rhea C.K., Silver T.A., Hong S.L., Ryu J.H., Studenka B.E., Hughes C.M., Haddad J.M. (2011). Noise and complexity in human postural control: Interpreting the different estimations of entropy. PLoS ONE.

[16] Kuznetsov N.A., Riley M.A. (2015). The role of task constraints in relating laboratory and clinical measures of balance. Gait Posture.

[17] Collins J.J., De Luca C.J. (1994). Upright, correlated random walks: A statistical-biomechanics approach to the human postural control system. Chaos.

[18] Collins J.J., De Luca C.J., Pavlik A.E., Roy S.H., Emley M.S. (1995). The effects of spaceflight on open-loop and closed-loop postural control mechanisms: Human neurovestibular studies on SLS-2. Exp. Brain Res..

[19] West B.J., Goldberger A.L. (1987). Physiology in Fractal Dimensions. Am. Sci..

[20] Lipsitz L.A., Goldberger A.L. (1992). Loss of complexity and aging. Potential applications of fractals and chaos theory to senescence. J. Am. Med. Assoc..

[21] Lipsitz L.A. (2002). Dynamics of Stability: The Physiologic Basis of Functional Health and Frailty. J. Gerontol..

[22] Goldberger A.L., Peng C.K., Lipsitz L.A. (2002). What is physiologic complexity and how does it change with aging and disease?. Neurobiol. Aging.

[23] Goldberger A.L., Rigney D.R., West B.J. (1990). Chaos and fractals in human physiology. Sci. Am..

[24] Task-Force (1996). Heart rate variability: Standards of measurement, physiological interpretation and clinical use. Task Force of the European Society of Cardiology and the North American Society of Pacing and Electrophysiology. Circulation.

[25] Shaffer F., Ginsberg J.P. (2017). An Overview of Heart Rate Variability Metrics and Norms. Front. Public Health.

[26] Stein P.K., Reddy A. (2005). Nonlinear heart rate variability and risk stratification in cardiovascular disease. Indian Pacing Electrophysiol..

[27] Godin P.J., Buchman T.G. (1996). Uncoupling of biological oscillators: A complementary hypothesis concerning the pathogenesis of multiple organ dysfunction syndrome. Crit. Care Med..

[28] Seely A.J., Christou N.V. (2000). Multiple organ dysfunction syndrome: Exploring the paradigm of complex nonlinear systems. Crit. Care Med..

[29] Novak V., Hu K., Vyas M., Lipsitz L.A. (2007). Cardiolocomotor coupling in yound and elderly people. J. Gerontol. Ser. A Biol. Sci. Med. Sci..

[30] Ferguson A.V., Latchford K.J., Samson W.K. (2008). The paraventricular nucleus of the hypothalamus—A potential target for integrative treatment of autonomic dysfunction. Expert Opin. Target..

[31] Giustina A., Veldhuis J. (1998). Pathophysiology of the Neuroregulation of Growth Hormone Secretion in Experimental Animals and the Human. Endocr. Rev..

[32] Wideman L., Consitt L., Patrie J., Swearingin B., Bloomer R., Davis P., Weltman A. (2006). The impact of sex and exercise duration on growth hormone secretion. J. Appl. Physiol..

[33] Hartman M.L., Veldhuis J.D., Thorner M.O. (1993). Normal control of growth hormone secretion. Horm. Res..

[34] Stein P.K., Kleiger R.E., Rottman J.N. (1997). Differing Effects of Age on Heart Rate Variability in Men and Women. Am. J. Cardiol..

[35] Agorastos A., Heinig A., Stiedl O., Hager T., Sommer A., Müller J.C., Schruers K.R., Wiedemann K., Demiralay C. (2019). Vagal effects of endocrine HPA axis challenges on resting autonomic activity assessed by heart rate variability measures in healthy humans. Psychoneuroendocrinology.

[36] Pulopulos M.M., Vanderhasselt M.A., De Raedt R. (2018). Association between changes in heart rate variability during the anticipation of a stressful situation and the stress-induced cortisol response. Psychoneuroendocrinology.

[37] Adlan A.M., Veldhuijzen van Zanten J., Lip G.Y.H., Paton J.F.R., Kitas G.D., Fisher J.P. (2018). Acute hydrocortisone administration reduces cardiovagal baroreflex sensitivity and heart rate variability in young men. J. Physiol..

[38] Nonell A., Bodenseh S., Lederbogen F., Kopf D., Hamann B., Gilles M., Deuschle M. (2005). Chronic but not acute hydrocortisone treatment shifts the response to an orthostatic challenge towards parasympathetic activity. Neuroendocrinology.

[39] Rodríguez-Liñares L., Vila X.A., Méndez A.J., Lado M.J., Olivieri D. R-HRV: An R-based software package for Heart Rate Variability analysis of ECG recordings. Proceedings of the 3rd Iberian Conference in Systems and Information Technologies.

[40] Yentes J.M., Hunt N., Schmid K.K., Kaipust J.P., McGrath D., Stergiou N. (2013). The appropriate use of approximate entropy and sample entropy with short data sets. Ann. Biomed. Eng..

[41] Pincus S. (1991). A regularity statistic for medical data analysis. J. Clin. Monit..

[42] Sassi R., Cerutti S., Lombardi F., Malik M., Huikuri H.V., Peng C.K., Schmidt G., Yamamoto Y. (2015). Advances in heart rate variability signal analysis: Joint position statement by the e-Cardiology ESC Working Group and the European Heart Rhythm Association co-endorsed by the Asia Pacific Heart Rhythm Society. Europace.

[43] Costa M., Goldberger A.L., Peng C.K. (2002). Multiscale entropy analysis of complex physiologic time series. Phys. Rev. Lett..

[44] Heffernan K.S., Fahs C.A., Shinsako K.K., Jae S.Y., Fernhall B. (2007). Heart rate recovery and heart rate complexity following resistance exercise training and detraining in young men. Am. J. Physiol. Heart Circ. Physiol..

[45] Kennel M.B., Brown R., Abarbanel H.D. (1992). Determining embedding dimension for phase-space reconstruction using a geometrical construction. Phys. Rev. A.

[46] Rhodes C., Morari M. (1997). The false nearest neighbors algorithm: An overview. Comput. Chem. Eng..

[47] Hussain V.S., Spano M.L., Lockhart T.E. (2020). Effect of data length on time delay and embedding dimension for calculating the Lyapunov exponent in walking. J. R. Soc. Interface.

[48] Theiler J., Eubank S., Longtin A., Galdrikian B., Farmer J.D. (1992). Testing for nonlinearity in time series: The method of surrogate data. Phys. D.

[49] Hurst H.E. (1956). The Problem of Long-Term Storage in Reservoirs. Int. Assoc. Sci. Hydrology. Bull..

[50] Weron R. (2002). Estimating long-range dependence: Finite sample properties and confidence intervals. Phys. A.

[51] Webber C.L., Zbilut J.P. (2005). Recurrence quantification analysis of nonlinear dynamical systems. Tutor. Contemp. Nonlinear Methods Behav. Sci..

[52] Coco M.I., Dale R. (2014). Cross-recurrence quantification analysis of categorical and continuous time series: An R package. Front. Psychol..

[53] Richman J.S., Moorman J.R. (2000). Physiological time-series analysis using approximate entropy and sample entropy. Am. J. Physiol. Heart Circ. Physiol..

[54] Zbilut J.P., Giuliani A., Webber C.L. (1998). Detecting deterministic signals in exceptionally noisy environments using cross-recurrence quantification. Phys. Lett. A.

[55] Shockley K., Butwill M., Zbilut J.P., Webber C.L. (2002). Cross recurrence quantification of coupled oscillators. Phys. Lett. A.

[56] R-Core-Team (2018). R: A Language and Environment for Statistical Computing.

[57] Kanaley J.A., Weltman J.Y., Veldhuis J.D., Rogol A.D., Hartman M.L., Weltman A. (1997). Human growth hormone response to repeated bouts of aerobic exercise. J. Appl. Physiol..

[58] Wideman L., Weltman J.Y., Hartman M.L., Veldhuis J.D., Weltman A. (2002). Growth hormone release during acute and chronic aerobic and resistance exercise. Sports Med..

[59] Weltman A., Weltman J.Y., Watson Winfield D.D., Frick K., Patrie J., Kok P., Keenan D.M., Gaesser G.A., Veldhuis J.D. (2008). Effects of continuous versus intermittent exercise, obesity, and gender on growth hormone secretion. J. Clin. Endocrinol. Metab..

[60] Buchman T.G. (2001). Multiple organ dysfunction syndrome. Surgery.

[61] Berry N.T., Wideman L., Rhea C.K. (2020). Variability and Complexity of Non-stationary Functions: Methods for Post-exercise HRV. Nonlinear Dyn. Psychol. Life Sci..

[62] Bonnemeier H., Weigand U.K.H., Brandes A., Kluge N., Katus H.A., Richardt G., Potratz J. (2003). Circadian Profile of Cardiac Autonomic Nervous Modulation in Healthy Subjects: Differing Effects of Aging and Gender on Heart Rate Variability. J. Cardiovasc. Electrophysiol..

[63] Berry N.T., Wideman L., Rhea C.K., Labban J., Chon K.H., Shykoff B.E., Haran F.J., Florian J.P. (2017). Effects of prolonged and repeated immersions of heart rate variability and complexity in military divers. Undersea Hyperb. Med..

[64] Weltman A., Weltman J.Y., Hartman M.L., Abbott R.D., Rogol A.D., Evans W.S., Veldhuis J.D. (1994). Relationship between age, percentage body fat, fitness, and 24-hour growth hormone release in healthy young adults: Effects of gender. J. Clin. Endocrinol. Metab..

[65] Kanaley J.A., Weatherup-Dentes M.M., Jaynes E.B., Hartman M.L. (1999). Obesity attenuates the growth hormone response to exercise. J. Clin. Endocrinol. Metab..

[66] Frystyk J. (2010). Exercise and the growth hormone-insulin-like growth factor axis. Med. Sci. Sports Exerc..

[67] Nindl B.C., Pierce J.R., Rarick K.R., Tuckow A.P., Alemany J.A., Sharp M.A., Kellogg M.D., Patton J.F. (2014). Twenty-hour growth hormone secretory profiles after aerobic and resistance exercise. Med. Sci. Sports Exerc..

[68] Hirako S., Wada N., Kageyama H., Takenoya F., Izumida Y., Kim H., Iizuka Y., Matsumoto A., Okabe M., Kimura A. (2016). Autonomic nervous system-mediated effects of galanin-like peptide on lipid metabolism in liver and adipose tissue. Sci. Rep..

[69] Fang P., He B., Shi M., Zhu Y., Bo P., Zhang Z. (2015). Crosstalk between exercise and galanin system alleviates insulin resistance. Neurosci. Biobehav. Rev..

[70] Tortorella C., Neri G., Nussdorfer G.G. (2007). Galanin in the regulation of the HPA (review). Int. J. Mollecular Med..

[71] Giustina A., Licini M., Schettino M., Doga M., Pizzocolo G., Negro-Vilar A. (1994). Physiological role of galanin in the regulation of anterior pituitary function in humans. Am. J. Physiol. Endocrinol. Metab..

[72] Sandoval-Alzate H.F., Agudelo-Zapata Y., Gonzalez-Clavijo A.M., Poveda N.E., Espinel-Pachon C.F., Escamilla-Castro J.A., Marquez-Julio H.L., Alvarado-Quintero H., Rojas-Rodriguez F.G., Arteaga-Diaz J.M. (2016). Serum Galanin Levels in Young Healthy Lean and Obese Non-Diabetic Men during an Oral Glucose Tolerance Test. Sci. Rep..

[73] Fang P., Shi M., Zhu Y., Bo P., Zhang Z. (2016). Type 2 diabetes mellitus as a disorder of galanin resistance. Exp. Gerontol..

[74] Mogharnasi M., TaheriChadorneshin H., Papoli-Baravati S.A., Teymuri A. (2019). Effects of upper-body resistance exercise training on serum nesfatin-1 level, insulin resistance, and body composition in obese paraplegic men. Disabil. Health J..

[75] Li Q.C., Wang H.Y., Chen X., Guan H.Z., Jiang Z.Y. (2010). Fasting plasma levels of nesfatin-1 in patients with type 1 and type 2 diabetes mellitus and the nutrient-related fluctuation of nesfatin-1 level in normal humans. Regul. Pept..

[76] Dore R., Levata L., Lehnert H., Schulz C. (2017). Nesfatin-1: Functions and physiology of a novel regulatory peptide. J. Endocrinol..

[77] Scotece M., Conde J., Abella V., Lopez V., Lago F., Pino J., Gomez-Reino J.J., Gualillo O. (2014). NUCB2/nesfatin-1: A new adipokine expressed in human and murine chondrocytes with pro-inflammatory properties, an in vitro study. J. Orthop. Res..

[78] Tanida M., Gotoh H., Yamamoto N., Wang M., Kuda Y., Kurata Y., Mori M., Shibamoto T. (2015). Hypothalamic Nesfatin-1 Stimulates Sympathetic Nerve Activity via Hypothalamic ERK Signaling. Diabetes.

[79] Oh I.S., Shimizu H., Satoh T., Okada S., Adachi S., Inoue K., Eguchi H., Yamamoto M., Imaki T., Hashimoto K. (2006). Identification of nesfatin-1 as a satiety molecule in the hypothalamus. Nature.

[80] Konczol K., Bodnar I., Zelena D., Pinter O., Papp R.S., Palkovits M., Nagy G.M., Toth Z.E. (2010). Nesfatin-1/NUCB2 may participate in the activation of the hypothalamic-pituitary-adrenal axis in rats. Neurochem. Int..

